# Unmasking Culprit Complex Infero-Posterior STEMI: Navigating Ad Hoc RCA CTO PCI

**DOI:** 10.1016/j.jaccas.2024.102798

**Published:** 2025-01-15

**Authors:** Maruf Sarwar, Stephen D. Adedokun, Keonmin Hwang, Mahesh Anantha Narayanan

**Affiliations:** a Section of Internal Medicine, White River Health, Batesville, Arkansas, USA; b Section of Cardiovascular Disease, University of Tennessee Health and Science, Memphis, Tennessee, USA; c Belmont University Thomas F. Frist College of Medicine, Nashville, Tennessee, USA; d Section of Cardiovascular Diseases, White River Health, Batesville, Arkansas, USA; e University of Arkansas Medical Sciences, Little Rock, Arkansas, USA

**Keywords:** ad hoc CTO PCI, advanced interventional techniques, chronic total occlusion, collateral circulation, coronary artery bypass graft, door-to-balloon time, dual angiography, graft occlusion, graft thrombosis, infero-posterior myocardial infarction, percutaneous coronary intervention, retrograde revascularization, reverse CART technique, right coronary artery, right posterolateral branch occlusion, saphenous vein graft occlusion, stenting, ST-segment elevation myocardial infarction, thrombectomy, tip-in technique

## Abstract

Patients presenting with acute coronary syndrome with ST-segment elevation myocardial infarction require rapid and decisive interventions to restore blood flow to the affected myocardium, minimizing ischemic damage. This case report is particularly unique because it involves a patient presenting with ST-segment elevation myocardial infarction, where the culprit lesion was an occluded coronary artery graft with an extensive thrombus burden. The complexity of this case necessitated a strategic shift to revascularize the chronically occluded native vessel instead of the graft. This highlights the intricate decision-making and advanced interventional techniques required in managing such complex clinical scenarios.

## Visual Summary

**Figure:**
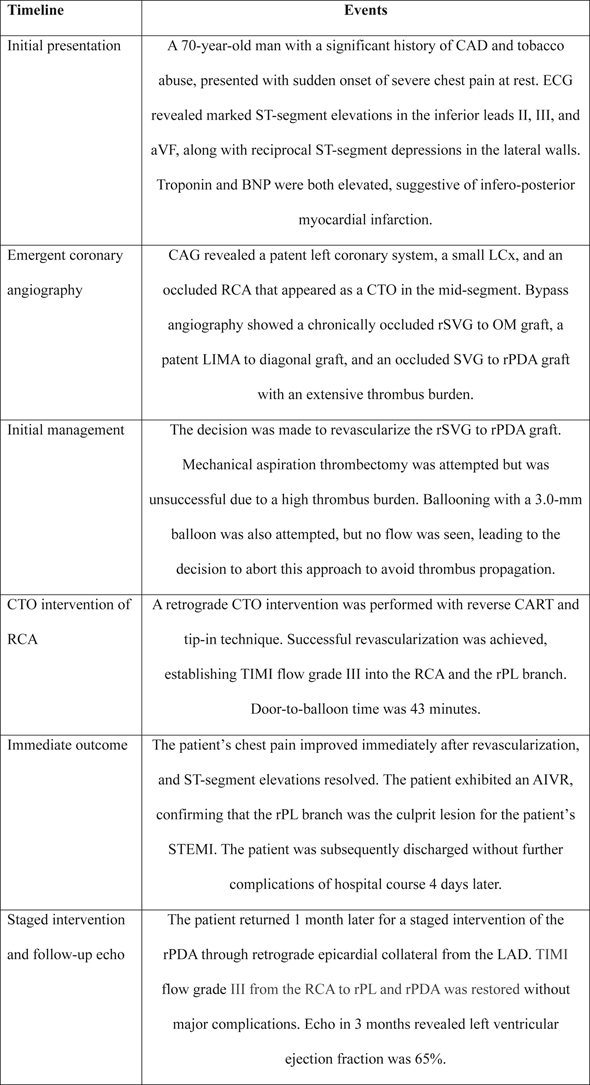

## History of Presentation

A 70-year-old man presented with sudden onset severe chest pain at rest. He had a significant history of tobacco abuse, smoking 1.5 packs per day. He was given aspirin en route to the hospital. On admission, he was afebrile, with a blood pressure of 132/72 mm Hg, pulse of 54 beats/min, and oxygen saturation of 94% on 2 L/min oxygen via nasal cannula. Physical examination was unremarkable.

**Learning Objectives**

• To understand the diagnostic challenges and therapeutic options available for ACS in patients with previously placed CABG requires a multifaceted approach.
• To evaluate the role of accurate ECG interpretation, emergent coronary angiography, and the decision-making process involved in identifying the culprit lesion is critical for interventional success.
• To analyze the indications for CTO intervention in the context of STEMI and the potential benefits and risks is paramount for optimal patient outcome.
• To assess the impact of successful CTO revascularization on patient outcomes, symptom resolution, and myocardial salvage in acute settings.

## Past Medical History

The patient had poorly controlled coronary artery disease with a previous myocardial infarction, requiring coronary artery bypass graft (CABG) 10 years prior (right saphenous vein graft [rSVG] to obtuse marginal, rSVG to right posterior descending artery [rPDA], and left internal mammary artery [LIMA] to the diagonal). He also experienced a cerebrovascular accident 12 years prior without major residual deficits. The patient was noncompliant with aspirin and high-intensity statin therapy. On social history, he had a history of alcohol abuse, consuming 3 to 4 beers daily.

## Differential Diagnosis

The differential diagnosis included acute coronary syndrome, aortic dissection, acute pericarditis, acute myocarditis, Prinzmetal angina, and Takotsubo cardiomyopathy.

## Investigations

**Figure 1:**
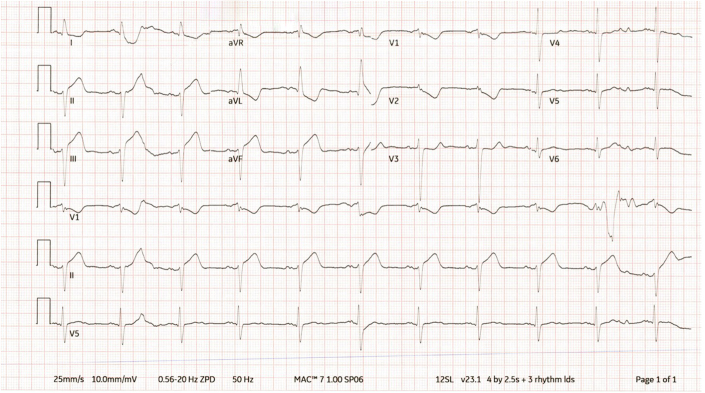
Presenting Electrocardiogram With ST-Segment Elevation Myocardial Infarction ST-segment elevations in the inferior leads and reciprocal changes in the anterolateral leads. Note the pattern of ST-segment depressions in V_1_ and V_2_, suggestive of posterior wall involvement.

Electrocardiogram (ECG) revealed marked ST-segment elevations in the inferior leads II and III, aVF with ST-segment elevation in lead II > III, along with reciprocal ST-segment depressions in the lateral walls, suggestive of infero-posterior myocardial infarction (IPMI) (Figure 1). Chest radiograph was normal. Initial chemistry and hematology panels were unremarkable, but troponin I (0.087 ng/mL) and brain natriuretic peptide (582.00 pg/mL) were elevated. The diagnosis of infero-posterior acute ST-segment elevation myocardial infarction (STEMI) was confirmed, and the cardiac catheterization laboratory was activated.

## Management

**Figure 2:**
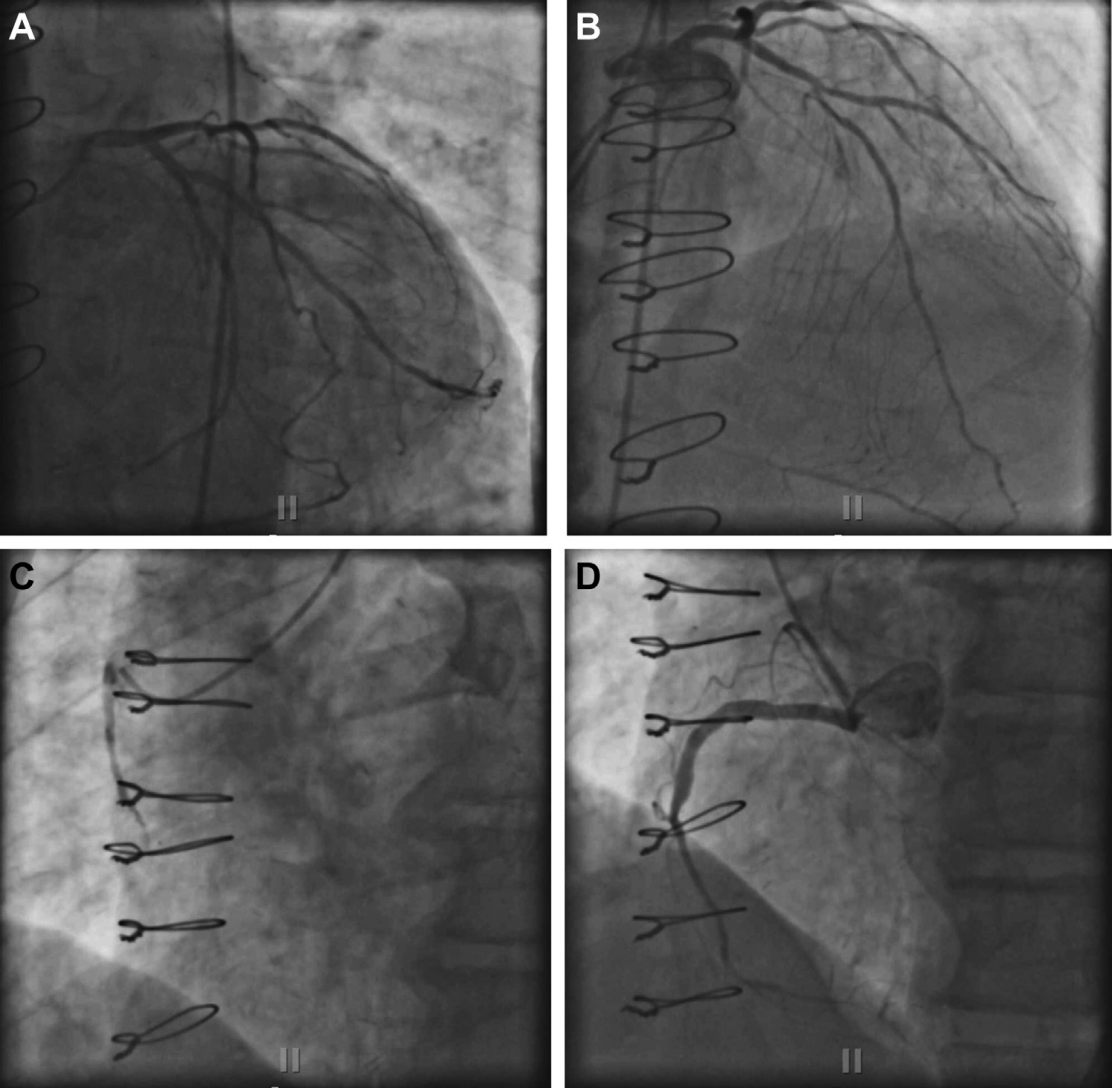
Diagnostic Angiogram With Bypass Angiography (A) Left coronary angiogram. Varying degrees of disease, with left main free of flow-limiting issues, mild disease in the left circumflex and the first obtuse marginal artery (30%), and diffuse stenosis in the left anterior descending artery (proximal 30%, mid 50%-60%, and distal 30%). (B) Patent left internal mammary artery. Diagonal branch filling patent bypass. Septal and epicardial branches filling the right posterior descending artery (rPDA) chronic total occlusion (CTO). (C) Occluded saphenous vein graft (SVG). Bypass angiography revealed extensive thrombus burden of the SVG to rPDA. (D) rPDA CTO. Right coronary artery is patent with stents, but the rPDA is chronically occluded and filled by collaterals from the left anterior descending artery, while a previous SVG to this area is also occluded.

Emergent coronary angiography revealed a patent left system with small left circumflex artery (LCx) and competing diagonal flow, and occluded right coronary artery (RCA) that appeared to be a chronic occlusion in the mid segment without significant bridging collaterals. Bypass angiography revealed a chronically occluded rSVG to obtuse marginal graft, patent LIMA to diagonal graft, and occluded saphenous vein graft (SVG) to rPDA graft with extensive thrombus burden, suggesting an acute presentation. Although the rPDA was a chronic total occlusion (CTO) past the anastomotic site of the rSVG with the rPDA, it was seen filling from the left anterior descending artery (LAD) septal and apical LAD epicardial collaterals, and thus seemed unlikely to be the culprit lesion (Figures 2A to 2D).

**Figure 3:**
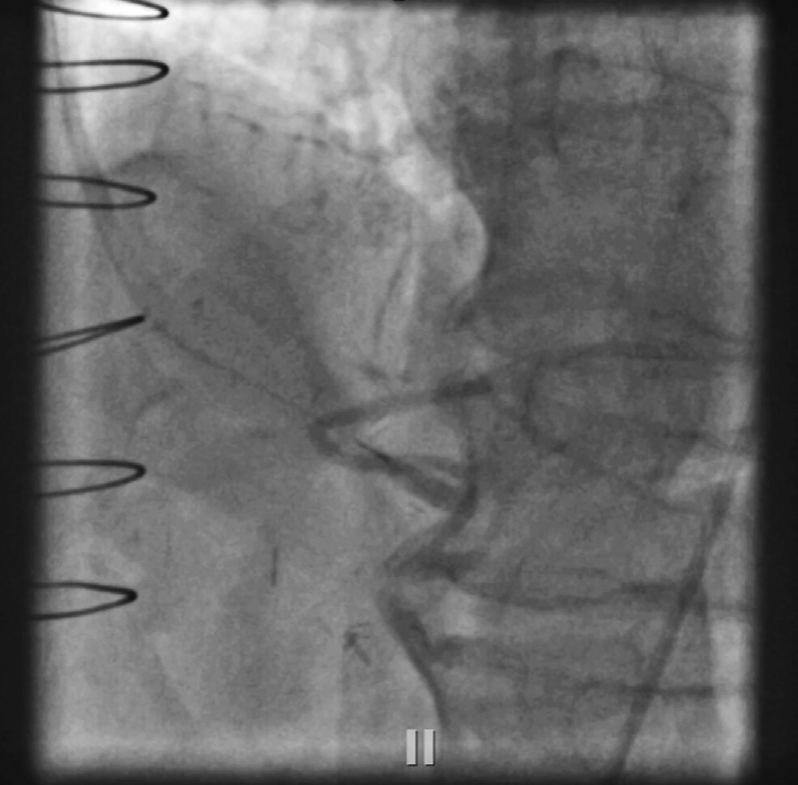
Native Right Coronary Artery Chronic Total Occlusion Up to the Bifurcation Microcatheter injection of distal right system showing a large trifurcated right posterolateral artery branch.

Because the LCx was not as large, we decided the culprit had to be a large right posterolateral artery (rPL) branch which was supplied retrograde from the rSVG to rPDA graft. Using a multipurpose guide to engage the graft, a polymer jacketed wire was then advanced retrograde into the bifurcation and a microcatheter injection was performed at the anastomotic site, which confirmed presence of a large trifurcated rPL branch and no filling of the distal RCA, suggesting the native RCA CTO extended up to the bifurcation (Figure 3, Video 1).

**Figure 4:**
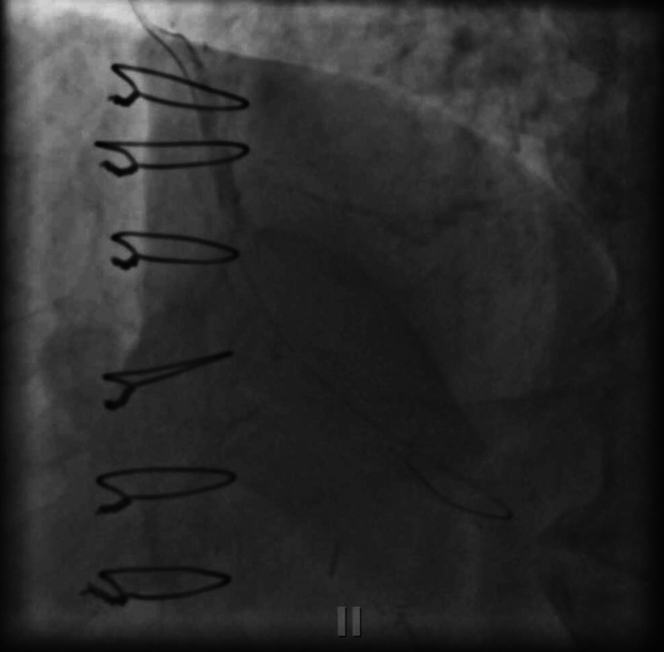
Attempted Mechanical Aspiration Thrombectomy Unsuccessful due to the extremely high thrombus burden.

Decision was made to revascularize the rSVG to rPDA graft. Aspiration thrombectomy and dilation with a 3.0-mm balloon failed to restore flow in the graft, leading us to abort further manipulations to avoid propagation of thrombus into the rPL (Figure 4). Filter wire was not considered given the lack of appropriate deployment zone. Quickly, we decided to proceed with CTO intervention of the RCA to be able to revascularize the rPL branch. The patient still had persistent ST-segment elevations and was complaining of worsening chest pain.

RCA was engaged antegrade with JR4 catheter. A retrograde microcatheter was advanced, entering the CTO cap at the distal RCA. Due to the ambiguous nature of the CTO cap and its location around a collateral, antegrade attempts were considered risky. We thought it was safer to proceed retrograde because we were already in the true lumen distally, and we had a microcatheter ready. The decision to use a retrograde approach minimized the risk of subintimal tracking and reentry after bifurcation, and it avoided compromising either branch of the bifurcation.

The rPDA was a CTO immediately after the anastomotic site of the graft, and it was filling retrograde. We were concerned that crossing the rPDA CTO retrograde could be challenging because the septal collaterals were exceedingly small, and we did not want to risk using an epicardial collateral during an emergency situation. Given these concerns, we opted for the vein graft due to the relative ease of wire navigation, which we thought was the safer and more effective approach at the time.

**Figure 5:**
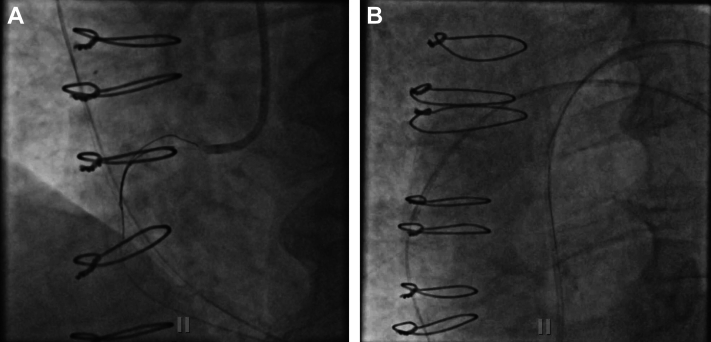
Ad Hoc Right Coronary Artery Chronic Total Occlusion Intervention With Reverse CART and Tip-In Technique (A) Reverse coronary artery revascularization therapy (CART) for chronic total occlusion. Performed reverse CART using an antegrade 3.5-mm balloon. (B) Tip-in technique. Reentered the true lumen antegrade with a Confianza wire using the tip-in technique.

**Figure 6:**
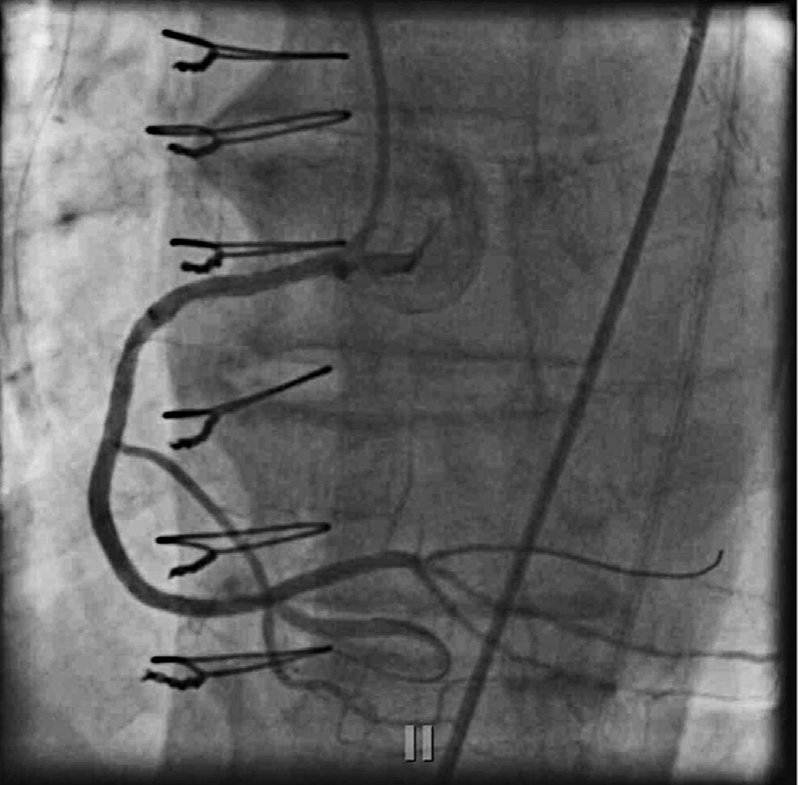
Successful Revascularization of RCA and rPL Chronic Total Occlusion The CTO lesion was predilated and stented from the mid to distal right coronary artery (RCA) into the right posterolateral artery (rPL) branch, resulting in TIMI flow grade III to the RCA and rPL trifurcation.

We then performed reverse coronary artery revascularization therapy (CART) with antegrade 3.5-mm balloon and reentered retrograde into the true lumen antegrade using Confianza (Asahi Intecc Medical) and performed tip-in, instead of externalization (Figures 5A and 5B, Video 2). Corsair (Asahi Intecc Medical) microcatheter was tracked on retrograde wire up to the bifurcation, and then we were able to wire into the rPL. Intravascular ultrasound was used before stenting to confirm that we were in the true lumen and to evaluate the vessel size for optimal stent placement. This helped ensure that the appropriate stent size was selected, contributing to the overall procedural success. The entire CTO lesion was predilated stented from mid to distal RCA into the rPL, which established TIMI flow grade III into the rPL branch (Figure 6, Video 3). Immediately after revascularization of the rPL branch, the patient’s chest pain improved, ST-segment elevations settled, and the patient had accelerated idioventricular rhythm, again confirming the rPL was the culprit. Our door-to-balloon time in this patient was 43 minutes.

## Discussion

IPMIs often have atypical presentation on standard 12-lead ECGs. These infarctions involve the inferior and posterior walls of the heart, areas typically supplied by the RCA or the LCx. One particularly unusual but noteworthy culprit for IPMIs is the rPL branch of RCA. The RPL branch can sometimes be responsible for supplying a significant portion of the infero-posterior myocardial region. When occluded, this branch can lead to an infarction that might be overlooked if clinicians focus solely on more common occlusion sites. Aung et al^1^ highlighted the potential importance of the rPL branch in unexplained posterior myocardial infarctions, documenting a case where its occlusion led to severe mitral regurgitation and complete heart block when typical suspect arteries (RCA or LCx) were unobstructed.^1^

Recognizing an IPMI caused by an rPL branch occlusion is crucial because its ECG manifestations may not align with the more familiar patterns associated with other coronary artery occlusions. Specifically, rPL branch occlusion may result in subtle or atypical changes, such as less pronounced ST-segment elevation in the inferior leads or isolated posterior lead involvement, often not noted in standard 12-lead ECGs.^2^ This underscores the necessity of using posterior leads (V_7_-V_9_) in suspected posterior infarctions and maintaining suspicion for less common culprits like the rPL branch.

CTOs are relatively common in patients with coronary artery disease, with a prevalence varying depending on the patient population. CTOs are found in up to 50% of patients undergoing coronary angiography.^3^ A study involving 14,439 patients at 3 Canadian centers reported an overall CTO prevalence of 18.2%.^3^, ^4^, ^5^ Among patients with at least one ≥50% stenosis, the prevalence of CTOs was 18.4%. Patients with prior CABG had a CTO prevalence of 54%. Although rare, it has been shown that patients with STEMI showed a 10% prevalence of CTOs.^3^ The presence of CTOs in the setting of STEMI presents a unique challenge, requiring careful consideration of the potential benefits of revascularization against the procedural risks.^6^

CTOs are frequently encountered in patients with a history of prior CABG, with incidence as high as 54%, because the native vessel tends to become a CTO with the prior existent bypass graft.^1^^,^^5^ About 50% of SVGs become occluded and 43% of bypassed native arteries develop CTOs within 1 year after CABG. Patients with prior CABG presenting with acute STEMI face worse angiographic outcomes and higher mortality, posing an increasing challenge. Percutaneous intervention (PCI) to a thrombotic occluded SVG yields suboptimal results compared with native coronary arteries due to high thrombus burden and no reflow risk.^7^

In our case, extensive graft thrombus prevented successful intervention, necessitating native vessel retrograde CTO revascularization through the occluded vein graft. This approach does not come without major complications. In addition to the risk of distal embolization, there is also an increased risk of systemic embolization and stroke during retrograde interventions through thrombotic vein grafts. However, retrograde approaches via an occluded SVG offer higher procedural success in complex or long-standing CTOs because the distal cap is typically softer and easier to penetrate than the more fibrotic or calcified proximal cap.^7^^,^^8^ Compared with antegrade interventions for acute total graft occlusions, retrograde native CTO interventions can also minimize the need for aggressive antegrade techniques, which carry a higher risk of complications (eg, vessel perforation) or suboptimal outcomes (eg, extensive antegrade dissection, reentry past bifurcation).^9^ This case underscores the importance of proficiency in some advanced interventional techniques, such as use of dual angiography, antegrade and retrograde revascularization techniques for CTO, and techniques such as tip-in to help avoid externalization, especially during such emergencies.

For interventionalists without the expertise in advanced CTO techniques, medical management to stabilize the patient and transfer to a specialized center can be a viable alternative. If a culprit lesion requiring CTO intervention is identified, timely transfer is crucial to ensuring the best patient outcomes. Additionally, ongoing education and training in CTO techniques can be highly beneficial in emergency scenarios. Published data on PCI for acute CTOs are limited, and ad hoc CTO PCI is generally not recommended. However, in cases of STEMI due to acute graft occlusion, native CTO PCI using the occluded graft as a retrograde conduit may be feasible if suitable. Our literature review identified only 4 cases where ad hoc CTO PCI was performed in STEMI with culprit bypass graft occlusion (Table 1), highlighting the rarity and clinical significance of this case.^7^^,^^10^ This case is unique in that the culprit lesion was a significantly occluded trifurcated rPL branch, making the diagnosis particularly challenging.

**Table 1:** Literature Review: Ad Hoc CTO Percutaneous Intervention Cases in STEMI With Occluded Grafts

	Patient 1^7^	Patient 2^7^	Patient 3^7^	Patient 4^8^	Patient 5^a^
Age at STEMI presentation, y	68	79	71	62	70
Sex	Female	Male	Female	Male	Male
Country	United Kingdom	United Kingdom	United Kingdom	Belgium	United States
History of CABG	Unknown	Unknown	20 y prior	1 y prior	10 y prior
STEMI on ECG	Inferior	Inferior	Inferior	Inferior	Infero-posterior
Culprit vessel	SVG supplying RCA Territory	SVG supplying RCA Territory	Reoccluded SVG supplying LCx territory	RITA graft supplying LCx and RCA	rPL supplied retrograde via rSVG through rPDA anastomosis
CTO vessel	RCA	RCA	In-stent CTO of LCx	RCA	RCA
Advanced interventional technique	Guideliner-assisted reverse CART	Guideliner-assisted reverse CART	Retrograde externalization	ECMO, guideliner-assisted reverse CART	Guideliner-assisted reverse CART, tip-in
Result	TIMI flow grade III	TIMI flow grade III	TIMI flow grade III	TIMI flow grade III	TIMI flow grade III
Length of stay, d	5	2	3	Not reported	4

CABG = coronary artery bypass graft; CART = coronary artery revascularization therapy; CTO = chronic total occlusion; ECG = electrocardiogram; ECMO = extracorporeal membrane oxygenation; LCx = left circumflex artery; RCA = right coronary artery; RITA = right internal thoracic artery; rPDA = right posterior descending artery; STEMI = ST-segment elevation myocardial infarction; SVG = saphenous vein graft.

a Representative of our case of occluded graft infero-posterior myocardial infarction requiring ad hoc CTO PCI of native vessel.

## Follow-Up

**Figure 7:**
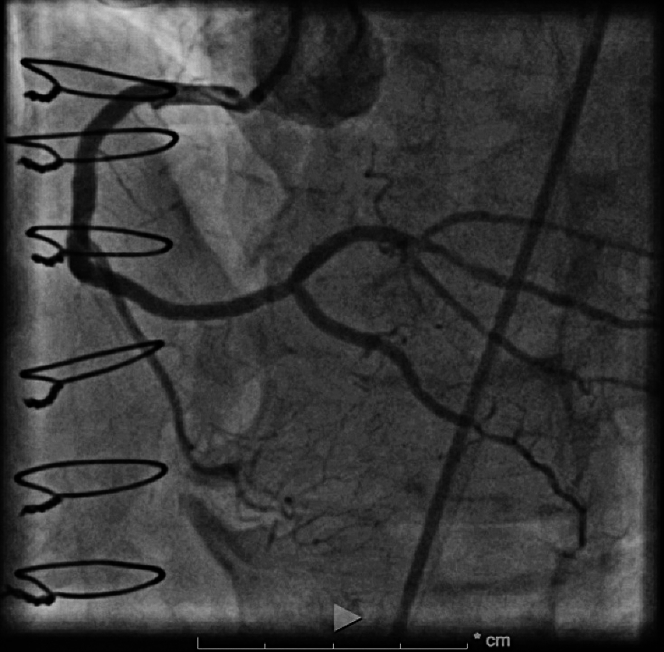
Follow-Up Staged Revascularization of rPDA CTO The final result of staged retrograde rPDA CTO revascularization. Final right coronary artery (RCA) angiography revealed TIMI flow grade III from the RCA to right posterolateral artery and rPDA. Abbreviations as in Figure 2.

The patient returned for a staged intervention of the rPDA through retrograde epicardial collateral from the LAD with no major complications (Figure 7, Video 4). During the follow-up, a diastolic hyperemia free ratio measurement of the mid LAD was performed, which was negative at 0.93. Because the stenosis was not flow-limiting, no intervention on the LAD was deemed necessary. The patient’s left ventricular ejection fraction was 65% at 3-month follow-up.

## Conclusions

This case highlights the complex decision-making in managing patients with STEMI with prior CABG and native vessel CTOs. It emphasizes the critical need for the broader interventional cardiology community to become proficient in advanced techniques because emergency scenarios require immediate and skilled intervention. Enhancing expertise and comfort with these advanced procedures ensures timely and effective treatment for all patients, particularly those who cannot afford delays in care. This collaborative growth will improve patient outcomes and elevate the standard of care in interventional cardiology.

**Visual Summary:** Timeline of Events

Timeline	Events
Initial presentation	A 70-year-old man with a significant history of CAD and tobacco abuse, presented with sudden onset of severe chest pain at rest. ECG revealed marked ST-segment elevations in the inferior leads II, III, and aVF, along with reciprocal ST-segment depressions in the lateral walls. Troponin and BNP were both elevated, suggestive of infero-posterior myocardial infarction.
Emergent coronary angiography	CAG revealed a patent left coronary system, a small LCx, and an occluded RCA that appeared as a CTO in the mid-segment. Bypass angiography showed a chronically occluded rSVG to OM graft, a patent LIMA to diagonal graft, and an occluded SVG to rPDA graft with an extensive thrombus burden.
Initial management	The decision was made to revascularize the rSVG to rPDA graft. Mechanical aspiration thrombectomy was attempted but was unsuccessful due to a high thrombus burden. Ballooning with a 3.0-mm balloon was also attempted, but no flow was seen, leading to the decision to abort this approach to avoid thrombus propagation.
CTO intervention of RCA	A retrograde CTO intervention was performed with reverse CART and tip-in technique. Successful revascularization was achieved, establishing TIMI flow grade III into the RCA and the rPL branch. Door-to-balloon time was 43 minutes.
Immediate outcome	The patient’s chest pain improved immediately after revascularization, and ST-segment elevations resolved. The patient exhibited an AIVR, confirming that the rPL branch was the culprit lesion for the patient’s STEMI. The patient was subsequently discharged without further complications of hospital course 4 days later.
Staged intervention and follow-up echo	The patient returned 1 month later for a staged intervention of the rPDA through retrograde epicardial collateral from the LAD. TIMI flow grade III from the RCA to rPL and rPDA was restored without major complications. Echo in 3 months revealed LV ejection fraction was 65% and normal right ventricular systolic function. There was impaired relaxation pattern of LV diastolic filling with mild concentric LV hypertrophy. No hypokinesis of the inferior wall was observed.

AIVR = accelerated idioventricular rhythm; BNP = brain natriuretic peptide; CAD = coronary artery disease; CAG = coronary angiography; CART = coronary artery revascularization therapy; CTO = chronic total occlusion; ECG = electrocardiogram; LCx = left circumflex artery; LIMA = left internal mammary artery; LV = left ventricular; OM = obtuse marginal; RCA = right coronary artery; rPL = right posterolateral artery; rSVG = right saphenous vein graft; STEMI = ST-segment elevation myocardial infarction.

## Funding Support and Author Disclosures

The authors have reported that they have no relationships relevant to the contents of this paper to disclose.

## Appendix

Video 1 Right Coronary Artery Chronic Total Occlusion Visualization Contrast injection via microcatheter reveals the distal right coronary system, highlighting a prominent trifurcated right posterolateral artery branch up to the point of bifurcation.

Video 2 Chronic Total Occlusion Recanalization Via Reverse Coronary Artery Revascularization Therapy Antegrade 3.5-mm balloon inflation facilitates reverse coronary artery revascularization therapy, creating a connection channel through the occlusion.

Video 3 Successful Revascularization of RCA and rPL Chronic Total Occlusion Balloon dilation and stenting from mid to distal RCA into rPL branch successfully reopens chronic blockage, achieving thrombolysis in myocardial infarction III flow to RCA and rPL trifurcation. RCA = right coronary artery; rPL = right posterolateral artery.

Video 4 Completing Right Coronary Artery Restoration: Follow-Up Right Posterior Descending Artery Intervention Final angiography shows thrombolysis in myocardial infarction III throughout the RC system after successful staged retrograde reopening of the right posterior descending artery chronic total occlusion.
